# Protective effect of snail secretion filtrate in an in vitro model of mastitis

**DOI:** 10.1002/vro2.70027

**Published:** 2026-02-15

**Authors:** Gianluca Antonio Franco, Ylenia Marino, Rosalia Crupi, Davide di Paola, Salvatore Cuzzocrea, Enrico Gugliandolo

**Affiliations:** ^1^ Department of Veterinary Science University of Messina Messina Italy; ^2^ Department of Chemical, Biological, Pharmaceutical and Environmental Science University of Messina Messina Italy; ^3^ Link University Rome Campus Italy

**Keywords:** bovine mastitis, inflammation, One Health, oxidative stress, snail secretion filtrate

## Abstract

**Background:**

Bovine mastitis is inflammation of the mammary gland mainly caused by bacterial infections, with relevant economic costs and implications related to antibiotic resistance. In light of the increasing demand for sustainable therapies, this study evaluated the anti‐inflammatory and antioxidant effects of snail secretion filtrate (SSF) from the species *Helix Aspersa Muller* in an in vitro model of bovine mastitis.

**Methods:**

Bovine mammary cells (MAC‐T) stimulated with lipopolysaccharide (LPS) were used to induce the inflammatory process. The cells were then treated with SSF to analyse its effects on oxidative stress, production of inflammatory cytokines (tumour necrosis factor‐α, interleukin‐6 and interleukin‐1β), expression of enzymes associated with inflammation such as inducible nitric oxide (iNOS) and cyclo‐oxygenase‐2 (COX‐2) and cytoprotection as heme‐oxygenase 1 (HO‐1).

**Results:**

Snail secretion filtrate significantly reduced the levels of reactive oxygen species and the production of pro‐inflammatory cytokines induced by LPS. Furthermore, it positively modulated the expression of iNOS and COX‐2, reducing their levels and increasing the expression of HO‐1, suggesting a cytoprotective action.

**Conclusions:**

Snail secretion filtrate was effective in reducing inflammation and oxidative stress in an in vitro model of bovine mastitis. The results suggest the potential use of SSF as a natural and sustainable remedy, in line with the One Health approach.

## INTRODUCTION

Bovine mastitis is one of the main diseases affecting dairy cattle, with significant repercussions on the health and wellbeing of the animal as well as on the quality of the products derived from it.^1^ Mastitis is an inflammation of the mammary gland of the bovine, mainly caused by bacterial infections by pathogens such as *Streptococcus agalactiae*, *Staphylococcus aureus*, *Corynebacterium bovis* and *Mycoplasma* spp., but it can also be caused by factors such as pathogens found in the environment in which the cows live, including *Klebsiella* spp., *Escherichia coli*, *Streptococcus uberis* and *Streptococcus dysgalactiae*.^2^ The udders of the cows act as a ‘reservoir’ for these pathogens, causing more or less prolonged infections of the mammary gland.^3^ The pathogenesis of this disease involves complex interactions between pathogens, the cow's immune system, and mammary epithelial cells, such as bovine primary mammary epithelial cells.^4^ The impact of bovine mastitis extends beyond the impairment of animal health and welfare, as it also compromises the quality and safety of dairy products such as milk. Thus, the disease also has a significant economic impact on the dairy industry due to reduced milk production and discarded milk, among other factors. The quality of this product is compromised, with an increase in the number of somatic cells and a reduction in its production due to the pathogens that infect the animal, one of all *S. agalactiae* or *S. dysgalactiae*.^5^

In addition, milk itself can act as a ‘reservoir’ for residues of antibiotics used in treatment, representing a challenge for food safety and the sustainability of dairy production. This therefore determines a significant challenge in terms of food safety and sustainability of dairy production.^6^ The emergence and spread of antimicrobial resistance (AMR) among mastitis‐causing pathogens is currently a growing concern in dairy production. Studies have shown that bacterial agents of bovine mastitis, such as *S. aureus*, *E. coli* and *Staphylococcus* spp., are increasingly resistant to common antibiotics used in this disease, including beta‐lactams, tetracyclines and macrolides. This resistance not only complicates clinical management of the disease, leading to higher rates of treatment failure, more prolonged infections and increased culling of affected animals, but also poses potential risks to public health, as resistant bacteria and antibiotic residues can enter the food chain through milk and dairy products. However, AMR is a global trend that is increasing, highlighting the urgent need for alternative therapeutic strategies and the prudent use of antimicrobials in veterinary medicine.^7^, ^8^, ^9^, ^10^, ^11^

To date, one of the most widely used therapeutic approaches to control inflammation in bovine mastitis relies on non‐steroidal anti‐inflammatory drugs (NSAIDs), such as flunixin meglumine, meloxicam and ketoprofen.^12^, ^13^, ^14^ These drugs primarily act on the cyclooxygenase enzyme, blocking the synthesis of prostaglandins and thromboxanes, which are potent mediators of pain, fever and oedema.^15^, ^16^ The use of NSAIDs is strongly recommended, particularly in cases of acute and/or severe mastitis with systemic symptoms. It is important to note that NSAIDs are commonly administered alone or in combination with antimicrobial therapy, as they lack antibacterial activity but provide supportive management by controlling the inflammatory response while the antimicrobials target the underlying infectious agent. This combined approach is therefore considered an effective strategy for optimising therapeutic outcomes in mastitic cows.

Although less common, corticosteroids, which act at a higher level of the inflammatory cascade by inhibiting phospholipase A2, have also been used in the past.^17^ However, their use is currently controversial because they have a strong immunosuppressive effect that can delay or hinder the complete elimination of the causative agent. In recent years, to mitigate the adverse side effects associated with conventional anti‐inflammatory therapies and to address concerns related to AMR, growing attention has been directed towards natural alternatives. In this context, the mucus secreted by the pedal glands of *Helix Aspersa Muller* has emerged as a promising candidate due to its documented biological activities.^18^, ^19^, ^20^

Traditionally used since ancient times for skin applications, snail secretion is currently employed in both the medical and cosmetic fields, particularly in wound care formulations.^21^ Snail mucus is a complex natural product characterised by the presence of hyaluronic acid, mucopolysaccharides, polyphenols and bioactive minerals.^19^ These components contribute synergistically to its functional properties. In particular, mucopolysaccharides and mucins, supported by the viscosity‐enhancing action of hyaluronic acid, promote strong adherence to biological surfaces, facilitating the formation of a protective barrier. Polyphenols and mineral elements further contribute by exerting antioxidant and tissue‐protective effects.^18^, ^22^, ^23^ Additionally, several constituents of snail secretion, including collagen, elastin, glycolic acid and allantoin, have been shown to exert reparative and anti‐inflammatory actions in different biological settings, such as gastric mucosal protection.^18^, ^24^, ^25^ Trace elements such as copper have also been implicated in tissue protection and healing processes.^26^, ^27^ Owing to these characteristics, the mucus extracted from *Helix Aspersa Muller* has been successfully used as a re‐epithelising treatment in the management of burn wounds in adult patients.^28^

Recent scientific evidence has underscored the specific efficacy of *Helix Aspersa Muller* mucus against the primary bacterial agents involved in the mastitis process. Unlike general pharmacological descriptions, research focusing on the peptides within this secretion has demonstrated a potent ability to inhibit biofilm formation by *S. aureus*, a critical factor in the persistence of mammary infections. Fractions of various molecular weights have shown low minimum inhibitory concentrations and the capacity to downregulate essential virulence factors, such as alpha‐haemolysin and coagulase, maintaining their effectiveness even against multidrug‐resistant strains. Furthermore, the demonstrated activity against *E. coli* and *S. agalactiae* confirms a broad‐spectrum potential that is particularly suited for addressing complex mammary pathologies.^29^, ^30^, ^31^, ^32^, ^33^ Implementing snail mucus as a therapeutic agent aligns with the One Health paradigm, offering a sustainable and biocompatible alternative for disease management. By prioritising such natural and non‐invasive solutions, it is possible to improve animal welfare while significantly reducing the clinical reliance on conventional anti‐inflammatory drugs and systemic antibiotics. This strategic shift is vital for mitigating the risk of AMR, a recurring global health priority.^8^, ^11^, ^34^, ^35^ In this context, the transition towards ‘green’ substances, including herbal therapies and immunological treatments, represents a modern frontier in veterinary medicine, providing effective recovery pathways without contributing to the further escalation of bacterial resistance.^35^, ^36^, ^37^, ^38^, ^39^, ^40^

Therefore, based on the known biological properties of snail slime and the physiopathology of mastitis, we hypothesised that treatment with snail secretion filtrate (SSF) may contribute to maintaining homeostasis and may represent a potential preventive or therapeutic strategy against mammary gland inflammation. Accordingly, the aim of this study was to evaluate the anti‐inflammatory and antioxidant effects of snail slime in an in vitro model of mastitis by assessing key elements involved in these processes, such as lipopolysaccharide (LPS)‐induced pro‐inflammatory cytokines (tumour necrosis factor‐α [TNF‐α], interleukin‐6 [IL‐6] and interleukin‐1β [IL‐1β]) and inflammatory enzymes such as cyclo‐oxygenase‐2 (COX‐2). In parallel, this study examined its ability to modulate reactive oxygen species (ROS) levels and other markers of oxidative stress, such as malondialdehyde (MDA), glutathione (GSH) and heme‐oxygenase 1 (HO‐1), in order to comprehensively evaluate its potential protective role in the context of mastitis.

## MATERIALS AND METHODS

### SSF chemical characterisation

The SSF used in this study was provided and characterised by Snail S.r.l. It was characterised by a high content of glycolic acid and collagen, with allantoin and elastin also present. A comprehensive summary of the constituent substances and spectrophotometric analysis, including sodium dodecyl sulphate‒polyacrylamide gel electrophoresis profiles of the SSF protein model and its electrophoretic characterisation, are detailed in the study by Gugliandolo et al.^18^

### SSF extraction

Snails were physically stimulated using the tip of a sterile cotton swab to extract the mucus mechanically. Next, it was passed through three separate filters (10, 1 and 0.22 µm; Pall) and stored at 4°C. To get rid of contaminants and endotoxins, the 0.22 µm filter is especially crucial.^18^

### Cell culture

The bovine mammary epithelial cell line (MAC‐T) was cultured using Dulbecco's minimum essential medium (CAT# D6429) supplemented with 10% fetal bovine serum and a standard antibiotic solution (200 U/mL streptomycin/penicillin, Sigma‒Aldrich, CAT# P4333). Cultures were maintained at 37°C in a humidified environment with a tension of 5% CO_2_, ensuring cell viability by changing the medium every 48 h. For experiments, cells were separated, once fully confluent, using a 0.25% trypsin solution; it was essential to use cells exclusively between passages 10 and 20, ensuring the absence of contamination and the preservation of typical epithelial morphology, strictly excluding all cultures that did not meet these quality criteria. Finally, to mimic physiological function, the in vitro lactation condition of MAC‐T cells was induced by the addition of a specific hormonal cocktail containing 1 µg/mL hydrocortisone, 5 µg/mL prolactin and 5 µg/mL insulin.^41^, ^42^, ^43^

### Cell treatment

The cells, which reached 90% confluence in a logarithmic growth phase, were exposed to LPS (1 µg/mL) and different concentrations of SSF (30%, 25%, 20%, 15%, 5% and 0.5%) for 24 h following previous protocols.^44^, ^45^, ^46^ Negative controls consisted of cells cultured in LPS (1 µg/mL) without SSF, while positive controls included LPS combined with 20 µg/mL dexamethasone (DEX).

### Cell viability assay

The possible toxic effect of SSF on MAC‐T cells was determined by methylthiazolyltetrazolium (MTT) assay selected in accordance with ISO 10993‐5, which identifies this colorimetric method based on mitochondrial metabolic activity as fully appropriate for the in vitro evaluation of cell viability. Briefly, 5 × 10^5^ cells were plated in a 96‐well plate and incubated with SSF at 30%, 25%, 20%, 15%, 5% and 0.5% for 24 h, followed by the MTT treatment (100 µL of 0.5 mg/mL each well) for 3 h. Dimethyl sulphoxide was added to dissolve any deposited formazan. The optical density at 550 nm was measured using a microplate reader and used to calculate the cell viability.

### Enzyme‐linked immunosorbent assay

Secretion of TNF‐α, IL‐6 and IL‐1β was measured using commercial enzyme‐linked immunosorbent assay (ELISA) kits from R&D Systems (bovine TNF‐α ELISA kit CAT# EBTNF, bovine IL‐6 uncoated ELISA kit CAT# ESS0029, bovine IL‐1β uncoated ELISA kit CAT# ESS0027). Briefly, the plates (Multiscreen 96‐well plate, solid bottom CAT# MSEHNFX40) were prepared with the coating containing the capture antibody for TNF‐α, IL‐6 and IL‐1β (100 µL) allowing the capture antibody diluted in the coating buffer to act overnight by adhering to the bottom of the plate. Subsequently, after two washes, the non‐specific antibody sites were blocked with 100 µL of diluent buffer (supplied by the kit) for 1 h. Then, 100 µL of samples (6 and 24 h) of standards (TNF‐α, IL‐6 and IL‐1β) was added to each well and incubated for 2 h at room temperature. After two washes, 100 µL of diluted detection antibody was added to each well and incubated for 2 h at room temperature. After two washes, 100 µL of the working dilution of streptavidin‒horseradish peroxidase (HRP) was added to each well and incubated for 30 min at room temperature. After washing, 100 µL of tetramethylbenzidine (TMB) substrate solution was added to each well and incubated for 15 min at room temperature in the dark. The reaction was then stopped by adding 100 µL of stop solution. The absorbance at 450 nm was recorded to calculate the cytokine concentration.

### Real‐time polymerase chain reaction

To evaluate the mRNA expression of target genes, RNA was extracted from MAC‐T cells using RNeasy kit (Qiagen, CAT# 74104), for real‐time polymerase chain reaction (RT‐PCR) analysis. Briefly, samples were first lysed and then ethanol was added to provide ideal binding conditions. The lysates were then loaded into the RNeasy silica membrane. RNA binds and all contaminants were efficiently washed away. The residual amounts of DNA remaining were removed using a convenient on‐column DNase treatment. Pure, concentrated RNA was eluted in 50 µL of water. iScript RT‐PCR kit (Bio‐Rad, CAT# 1708890) was used to synthesise first‐strand cDNA. Briefly, the reverse transcription master mixture was prepared by adding 1 µg of RNA template (iScript RT Supermix; Bio‐Rad, CAT# 1708840) and nuclease‐free water. The complete reaction mixture was incubated in a thermal cycler (priming 5 min at 25°C, reverse transcription 20 min at 46°C and RT inactivation for 1 min at 95°C). Real‐time PCR analysis was performed by SYBR Green method on a real‐time PCR system (Applied Biosystems). Polymerase chain reaction conditions were as follows: initial denaturation at 95°C for 15 min, followed by 45 cycles of amplification at 95°C for 20 s and 60°C for 40 s. Final extension at 60°C for 60 s and a hold at 4°C were then performed. Data analysis was performed using the 2−∆∆Ct (2‒delta delta Ct) method, in which relative mRNA expression of target mRNAs (iNOS, HO‐1 and COX‐2) was compared to that of a constitutively expressed gene (i.e., GAPDH). The sequences of the specific primers, designed for bovine (*Bos taurus*) sequences, were as follows: for iNOS, forward 5′‐CCTAGCAGAAACCAAAGACAAG‐3′ and reverse 5′‐CAACATTATTCCTTGCAGACTTC‐3′; for COX‐2, forward 5′‐ACAGACATCACATCATTCCCAG‐3′ and reverse 5′‐ATGGTCAGCAAGACGTTTGAC‐3′; for HO‐1, forward 5′‐CCCTGCTCGTACTTTCAGAAG‐3′ and reverse 5′‐TGGCATAAACTTGCCCTACTG‐3′; and for the reference gene GAPDH, forward 5′‐TGACATTGAAACCATCACTGCC‐3′ and reverse 5′‐TGTAGGCCATGAGGTCCACC‐3′.

### Flow cytometry

2′,7′‐Dichlorodihydrofluorescein diacetate (H2DCFDA) dye (Sigma‒Aldrich, CAT# 287810) was used for total ROS level measurement with reference to the instructions from the manufacturer. MAC‐T cells (5000 cells/well in concentration) were plated in a 96‐well microplate and processed as indicated. Next, the probe (10 µmol/L)‐loaded cells were placed for 30 min away from light (37°C) and gently cleaned three times in phosphate‐buffered saline. Total ROS were evaluated using flow cytometry analysis and expressed as a variation over control of the mean fluorescence intensity.

### Assessment of oxidative stress and prostaglandin E_2_

Oxidative marker levels were assessed by evaluating the levels of markers such as MDA, the antioxidant counterpart GSH and prostaglandin E_2_ (PGE_2_).^47^ For PGE_2_ values, the PGE_2_ parameter ELISA kit KGE004B was used.

### Griess assay

The Griess reaction was used to measure nitrite levels. In a 96‐well plate, cells were plated at a density of 4 × 10^4^ cells/well and exposed to different concentrations of SSF (15%, 5% and 0.5%) for a full day. The supernatant of each sample was collected and subjected to the Griess reaction. Specifically, 50 µL of supernatant and 50 µL of 1% sulphanilamide solution (in 5% phosphoric acid) were added to another 96‐well plate. After a 10 min incubation period, each well received 50 µL of a 0.1% N‐1‐naphthylethylenediaminedihydrochloride solution, which was then incubated for another 10 min. A plate reader was used to measure the absorbance at 540 nm over 30 min.^47^

### Statistical analysis

For each experiment, three independent experiments were performed, and for each experiment, five repeat samples were used. The data resulting from all experiments are expressed as means ± standard error of the mean. The data were tested for normality and log‐normality using the Shapiro–Wilk test and, upon confirmation, treated as parametric. Statistical differences between groups were compared using ANOVA. A *p*‐value of less than 0.05 was considered statistically significant.

## RESULTS

### Effect of SSF on MAC‐T cell viability

**FIGURE 1:**
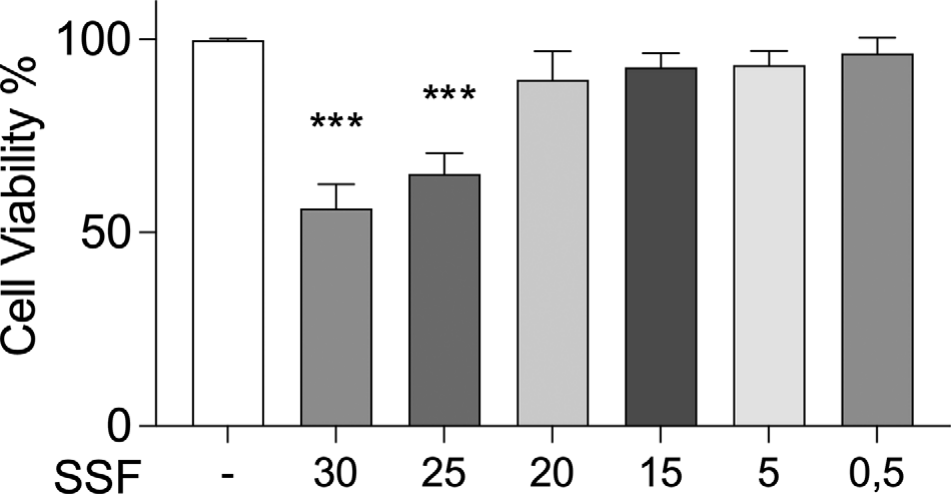
Effect of snail secretion filtrate (SSF) on MAC‐T cell viability by methylthiazolyltetrazolium assay. Data represent at least five experiments and are presented as the means ± standard error of the mean. ^***^ *p* < 0.001 versus control.

The cytotoxic potential of SSF on the MAC‐T cell line was evaluated using the MTT cell viability assay. The concentrations we evaluated were 30%, 25%, 20%, 15%, 5% and 0.5%. The cells were incubated for 24 h with SSF and then treated with MTT (100 µL of 0.5 mg/mL) for 3 h. As shown in Figure 1, no significant differences were revealed at 20, 15%, 5% and 0.5% compared to the control. However, a significant mortality was shown at the higher doses (30% and 25%) compared to the control. The dose of 20% did not show significant mortality, but since a discrete cell death was nevertheless evidenced, we decided to focus on 15%, 5% and 0.5%.

### Effect of SSF on oxidative stress LPS induced on MAC‐T cells

To evaluate the protective effect of SSF on MAC‐T cells exposed to the pro‐oxidant action of LPS, we co‐treated the cells with LPS (1 µg/mL) and SSF (15%, 5% and 0.5%) for a period of 6 and 24 h.

**FIGURE 2:**
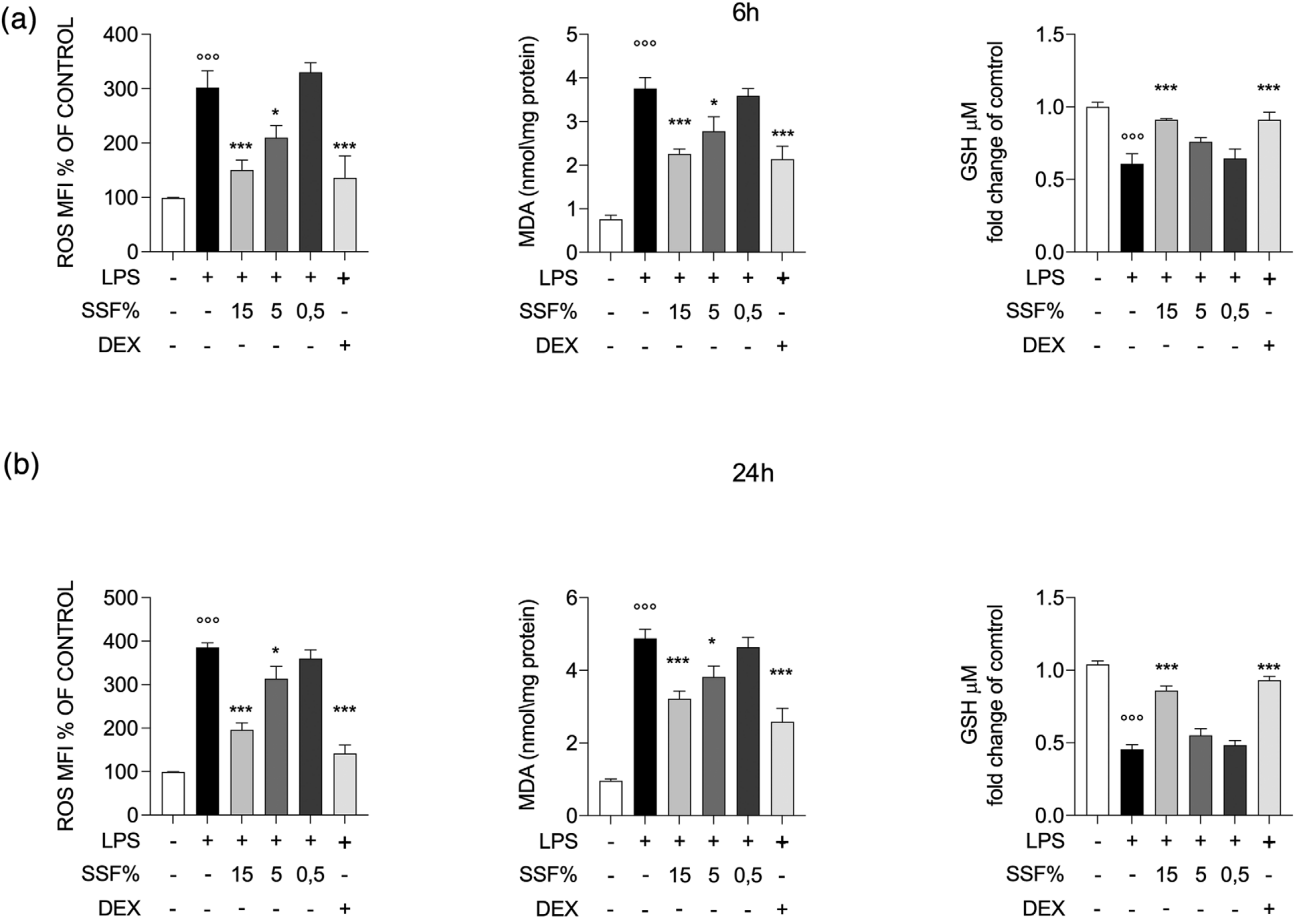
Effect of snail secretion filtrate (SSF) on oxidative stress and antioxidant markers at 6 h (a) and 24 h (b) after lipopolysaccharide (LPS) induction. Data represent at least five experiments and are presented as the means ± standard error of the mean. ^*^ *p* < 0.05, ^**^ *p* < 0.001, ^°°°^ *p* < 0.001 versus control. DEX, dexamethasone; GSH, glutathione; MDA, malondialdehyde; MFI, mean fluorescent intensity; ROS, reactive oxygen species.

After 6 h of exposure (Figure 2a), a marked increase in oxidative stress was observed, evidenced by a significant increase in the levels of ROS and MDA, accompanied by a reduction in GSH, the main endogenous antioxidant. In the groups subjected to co‐exposure with LPS and SSF, the protective effect of snail mucus was concentration dependent. In particular, the 15% concentration determined a significant reduction in the production of ROS and MDA, with an effect comparable to that of DEX, used as a positive control. The 5% concentration also showed a protective activity, although less marked than that observed at the highest concentration, while the 0.5% SSF did not produce significant differences compared to cells treated with LPS alone. As for GSH, only the treatment with 15% SSF preserved the levels of this endogenous antioxidant, with an effect comparable to the positive control. After 24 h (Figure 2b) from the beginning of the treatment, the results obtained confirmed what was observed at 6 h, with a significant reduction in oxidative stress in cells treated with the highest concentrations of SSF, with a more evident effect for the group treated with 15% SSF.

**FIGURE 3:**
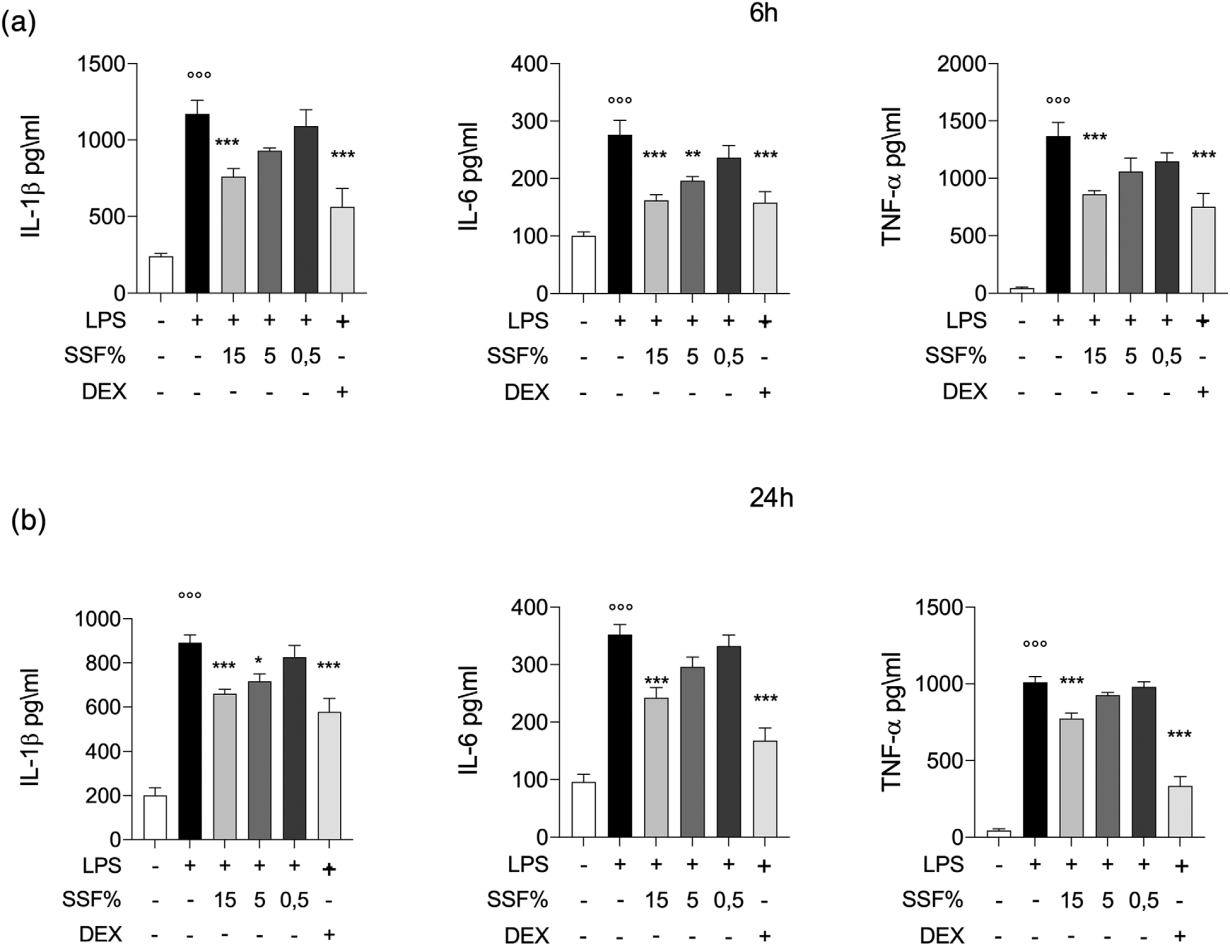
Effect of snail secretion filtrate (SSF) on tumour necrosis factor‐α (TNF‐α), interleukin‐6 (IL‐6) and interleukin‐1β (IL‐1β) secretion by MAC‐T cells at 6 h (a) and after 24 h (b) from lipopolysaccharide (LPS) induction. Data represent at least five experiments and are presented as the means ± standard error of the mean. ^**^ *p* < 0.005, ^***^ *p* < 0.001, ^°°°^ *p* < 0.001 versus control. DEX, dexamethasone.

We studied the effect of SSF on the release of pro‐inflammatory cytokines IL‐6, IL‐1β and TNF‐α in MAC‐T cells, as shown in Figure 3. Snail secretion filtrate 0.5% had no significant effect, as cells continued to produce cytokines in amounts similar to those treated with LPS alone, both after 6 h and after 24 h. Snail secretion filtrate 15%, instead, significantly reduced the release of all cytokines, both after 6 h and after 24 h. Snail secretion filtrate 5% had a partial effect, determining only the reduction in IL‐6 after 6 h, while after 24 h, it lowered the levels of IL‐1β and TNF‐α.

### Effect of SSF on iNOS, COX‐2 and HO‐1 expression

**FIGURE 4:**
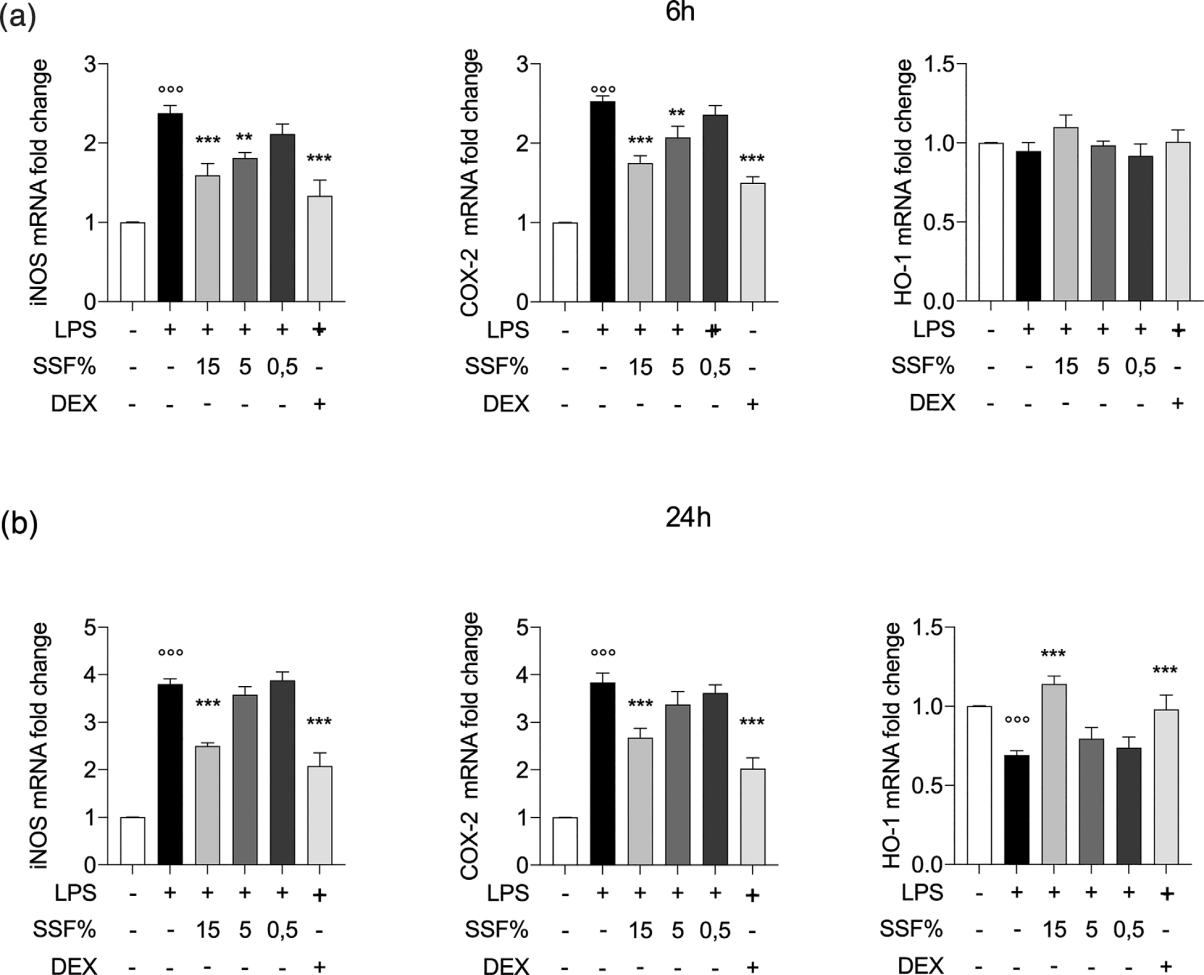
Effect of snail secretion filtrate (SSF) on iNOS, cyclo‐oxygenase‐2 (COX‐2) and heme‐oxygenase 1 (HO‐1) expression after 6 h (a) and after 24 h (b) from lipopolysaccharide (LPS) induction. Data represent at least five experiments and are presented as the means ± standard error of the mean. ^**^ *p* < 0.005, ^***^ *p* < 0.001, ^°°°^ *p* < 0.001 versus control. DEX, dexamethasone.

To better evaluate the anti‐inflammatory and antioxidative effect of SSF on LPS‐induced inflammation, we evaluated the mRNA expression levels of iNOS, COX‐2 and HO‐1, as well as their corresponding functional readouts, nitric oxide (NO) and PGE_2_, which are key mediators of inflammatory and oxidative processes. Figure 4a shows that at 6 h after exposure, SSF can significantly reduce the mRNA expression levels of iNOS and COX‐2 at 15% and 5%. Regarding HO‐1, a non‐significant increase compared to control was shown in HO‐1 mRNA levels in the SSF 15% group. However, at 24 h (Figure 4b), 15% SSF positively modulated the mRNA levels of iNOS, COX‐2 and HO‐1, making them comparable to those observed in the positive control DEX. In contrast, SSF concentrations of 5% and 0.5% showed no significant changes.

**FIGURE 5:**
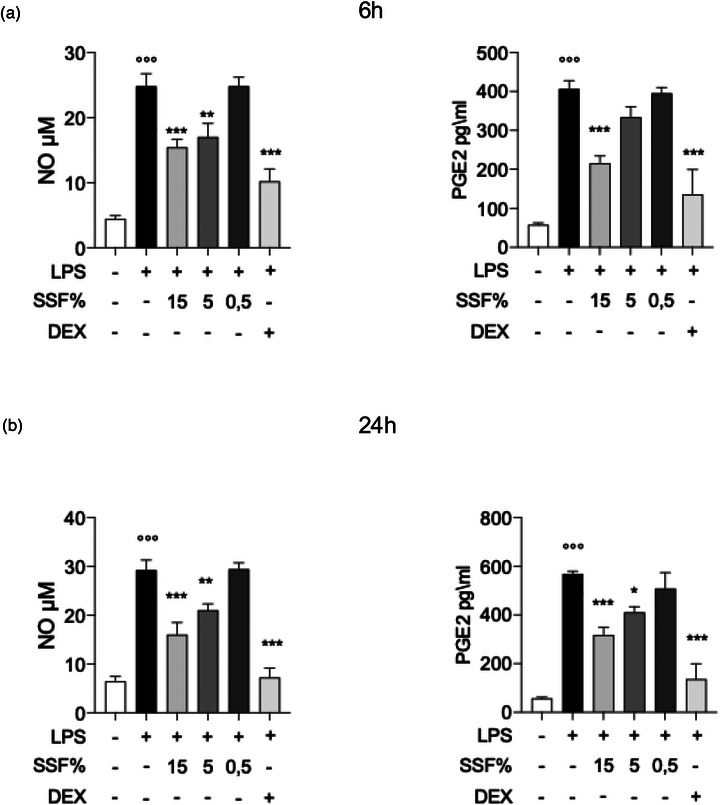
Effect of snail secretion filtrate (SSF) on nitric oxide (NO) and prostaglandin E2 (PGE_2_) release in lipopolysaccharide (LPS)‐stimulated MAC‐T cells after 6 h (a) and after 24 h (b). NO and PGE_2_ levels were quantified in culture supernatants at 6 and 24 h following LPS stimulation. Data represent at least five experiments and are presented as the means ± standard error of the mean. ^**^ *p* < 0.005, ^***^ *p* < 0.001, ^°°°^ *p* < 0.001 versus control. DEX, dexamethasone.

Consistent with the mRNA data, SSF also attenuated the release of NO and PGE_2_, two functional downstream mediators of iNOS and COX‐2 activity, respectively. At 6 h (Figure 5), LPS markedly increased both NO and PGE_2_ production, while SSF 15% significantly reduced NO levels and strongly suppressed PGE_2_ release. Snail secretion filtrate 5% showed a partial but measurable inhibitory effect, whereas SSF 0.5% was ineffective. At 24 h (Figure 5), SSF 15% maintained a robust inhibition of NO and PGE_2_ secretion, reaching values comparable to DEX. These functional results support the transcriptional findings and confirm that SSF dampens both the inflammatory (iNOS/NO and COX‐2/PGE_2_) and the antioxidative (HO‐1) molecular pathways activated by LPS.

## DISCUSSION

The aim of our research was to investigate the effect of *Helix Aspersa Muller* mucus in an in vitro model of bovine mastitis and to evaluate the anti‐inflammatory and antioxidant effect of it. The choice of treatment depends on several key factors, including the time from the onset of infection, the level of inflammation, the presence of pain, the chronicity of the condition and the severity of the lesions.^48^, ^49^, ^50^ In accordance with this approach, our study explored the therapeutic potential of *Helix Aspersa Muller* mucus in an in vitro model of bovine mastitis using a typical cell line as bovine mammary epithelial MAC‐T cells.^33^ We first determined the non‐cytotoxic SSF concentrations for the in vitro model, using dosages reported in the literature (30%, 25%, 20%, 15%, 5% and 0.5%). The results highlighted a significant cytotoxicity for the 30% and 25% concentrations, while the 20% concentration, although not lethal, showed mild toxic effects. For this reason, we selected 15%, 5% and 0.5% concentrations for the subsequent analyses, in accordance with the literature.^19^

Inflammation and oxidative stress play a crucial role in the immune response during mastitis. Pathogens activate Toll‐like receptors on epithelial cells, triggering intracellular signalling, specifically the nuclear factor‐kappa B (NF‐κB) pathway, which results in the production of ROS and pro‐inflammatory cytokines such as TNF‐α, IL‐6 and IL‐1β.^51^, ^52^, ^53^ In this context, our results have highlighted how SSF determines a reduction in intracellular ROS, thus suggesting a protective effect against oxidative damage.^54^, ^55^ In addition, our results highlight how SSF also determines a reduction in the levels of MDA, the final product of membrane fatty acid peroxidation, a process typical of oxidative stress in inflammatory conditions. Conversely, SSF can determine the maintenance of the levels of GSH, an essential tripeptide involved in antioxidant processes, keeping them close to physiological values.^56^, ^57^ Furthermore, SSF also has a significant effect on a key enzyme involved in oxidative processes, HO‐1. Heme‐oxygenase 1 determines the degradation of heme, a pro‐oxidant molecule present in red blood cells and other heme‐containing proteins. Through its metabolic products, HO‐1 performs various functions, including the antioxidant one. Our results demonstrate how SSF 15% causes a significant increase in this enzyme.^58^

In addition to causing cellular damage, oxidative stress is closely related to the activation of the inflammatory response.^59^ In fact, the accumulation of ROS and the balance of pro‐oxidant molecules can stimulate the production of pro‐inflammatory cytokines, amplifying the inflammatory process and contributing to tissue damage.^60^ In this context, the ability of SSF to reduce oxidative stress could also have a significant impact on the modulation of the inflammatory response.

Cytokines play a crucial role in inflammatory processes. Tumour necrosis factor‐α is recognised as a potent activator of the inflammatory process by inducing the expression of adhesion molecules and the production of other pro‐inflammatory cytokines.^61^ Interleukin‐1β instead has several functions to promote inflammation, including the activation of immune cells and the production of inflammatory prostaglandins.^62^ Interleukin‐6 is a cytokine produced by mammary epithelial cells, contributing to the local immune response.^63^ Our results highlight how SSF allows to determine a reduction in the secretion of TNF‐α, IL‐1β and IL‐6, consequently positively modulating the inflammatory process.^64^

Cytokines such as TNF‐α, IL‐1β and IL‐6 can stimulate the activation of key enzymes, amplifying the inflammatory process and oxidative stress. Among these, the enzyme iNOS catalyses the production of NO, which reacts with superoxide to form peroxynitrite, a highly toxic molecule with pro‐oxidative effects.^65^, ^66^ The results showed a reduction of this in the presence of SSF, suggesting a reduction in the production of NO and the oxidative stress associated with it. Heme‐oxygenase 1 is instead an enzyme with anti‐inflammatory and cytoprotective effects, capable of degrading heme into biliverdin, iron and carbon monoxide, compounds that have antioxidant activity.^67^, ^68^ Also, in this case SSF has a modulatory action, increasing the expression of HO‐1, suggesting a mechanism of cellular protection. Cyclo‐oxygenase‐2 is an enzyme involved in the synthesis of inflammatory prostaglandins, as well as responsible for pain sensitisation and maintenance of the inflammatory response.^16^ Snail secretion filtrate determined a reduction in the expression of COX‐2, thus suggesting a positive modulation of both inflammation and pain derived from it to produce prostaglandins related to it. The functional data on NO and PGE_2_ production further support the transcriptional findings and strengthen the evidence that SSF exerts a tangible anti‐inflammatory effect in LPS‐stimulated MAC‐T cells. As expected, LPS markedly increased NO and PGE_2_, reflecting the activation of the iNOS and COX‐2 pathways, two key drivers of bovine mammary inflammation. Snail secretion filtrate at 15% significantly attenuated both mediators at 6 and 24 h, closely mirroring the downregulation of iNOS and COX‐2 mRNA and reaching values comparable to the reference anti‐inflammatory compound DEX. This concordance between gene expression and functional output indicates that SSF not only modulates transcription but also effectively reduces the downstream enzymatic activity associated with the inflammatory cascade.

Our findings are in agreement with previous studies investigating natural therapies for mastitis. For example, plant‐derived compounds and bioactive peptides have shown similar anti‐inflammatory and antioxidant effects in bovine mammary epithelial cells or in small animal models of mastitis.^2^, ^38^, ^69^, ^70^, ^71^, ^72^, ^73^ These comparisons strengthen the potential relevance of snail slime as an alternative, supporting its efficacy and positioning it within the broader context of sustainable and One Health therapeutic strategies.

Although our in vitro results on MAC‐T cells have given promising results regarding the anti‐inflammatory and antioxidant effect of SSF, and several studies discuss the activity and safety of snail slime in vivo on mice and rats, it is important to recognise that translation into in vivo studies requires careful evaluation.^18^, ^20^, ^74^, ^75^ Future investigations should include evaluating the safety profile of snail slime in cattle by carefully determining effective dosages and choosing appropriate routes of administration for the mammary gland. Furthermore, it will be necessary to evaluate pharmacokinetic and pharmacodynamic parameters to ensure that the biologically active components reach their targets at therapeutic concentrations. These studies will be essential to validate the potential of snail slime as a new treatment for bovine mastitis in a physiological context.

Our findings are consistent with previous studies investigating therapies for mastitis. Plant‐derived compounds and bioactive peptides have shown similar anti‐inflammatory and antioxidant effects in bovine mammary epithelial cells, strengthening the potential relevance of snail slime as a sustainable and effective alternative.^76^, ^77^, ^78^, ^79^, ^80^ However, the implications of this study extend beyond the management of bovine mastitis. From a broader One Health perspective, the ability of snail slime to modulate highly conserved signalling pathways, such as the NF‐κB cascade and the COX‐2/iNOS axis, suggests that its therapeutic potential could be explored in other inflammatory pathologies across different species. Given the physiological similarities in the inflammatory response across mammals, these findings offer a promising proof of concept for the use of *Helix Aspersa Muller* secretions in the treatment of mucosal or epithelial inflammation in other livestock or even domestic animals. Furthermore, while any application in human medicine should be approached with caution, the biocompatibility and regenerative properties of snail slime suggest potential future applications in human inflammatory disorders or wound management. If its in vivo safety and efficacy are confirmed, snail slime could represent a sustainable bridge between veterinary and human clinical research, contributing to the global effort to reduce reliance on antibiotics through multi‐targeted natural bioactive compounds. These prospects underscore the importance of snail slime not only as a topical treatment for cattle, but also as a valuable resource in the development of interspecies therapeutic strategies.

## CONCLUSIONS

In conclusion, our findings demonstrate the efficacy of SSF in mitigating the inflammatory response in bovine MAC‐T cells by suppressing oxidative stress and modulating key enzymatic and cytokine pathways. While the MAC‐T model serves as a robust surrogate for mammary tissue, these preliminary anti‐inflammatory and antioxidant results necessitate further validation in animal models to confirm safety and performance within a complex physiological environment. Such steps are essential for developing translational applications that align with the One Health paradigm. Ultimately, this study positions SSF as a promising, sustainable strategy for managing bovine mastitis, opening innovative avenues for natural therapeutic interventions in veterinary medicine.

## AUTHOR CONTRIBUTIONS

*Conceptualisation*: **Enrico Gugliandolo**. *Formal analysis*: **Rosalia Crupi**. *Investigation*: **Gianluca Antonio Franco** and **Ylenia Marino**. *Resources*: **Salvatore Cuzzocrea**. *Writing—original draft preparation and writing—review and editing*: **Gianluca Antonio Franco**. *Methodology*: **Davide di Paola**. All the authors have read and agreed to the published version of the manuscript.

## CONFLICTS OF INTEREST

The authors declare they have no conflicts of interest.

## FUNDING INFORMATION

The authors received no specific funding for this work.

## ETHICS STATEMENT

The authors confirm that the ethical policies of the journal, as noted on the journal's author guidelines page, have been adhered to. No ethical approval was required as no animals were used.

## Data Availability

All the data generated are presented in this manuscript
